# Specific targeting of proteins to outer envelope membranes of endosymbiotic organelles, chloroplasts, and mitochondria

**DOI:** 10.3389/fpls.2014.00173

**Published:** 2014-04-29

**Authors:** Junho Lee, Dae Heon Kim, Inhwan Hwang

**Affiliations:** ^1^Cellular Systems Biology, Department of Life Sciences, Pohang University of Science and TechnologyPohang, South Korea; ^2^Division of Integrative Biosciences and Bioengineering, Pohang University of Science and TechnologyPohang, South Korea

**Keywords:** AKR2, β-barrel proteins, chloroplasts, endosymbiotic organelles, mitochondria, outer membrane proteins, signal-anchored proteins, tail-anchored protein

## Abstract

Chloroplasts and mitochondria are endosymbiotic organelles thought to be derived from endosymbiotic bacteria. In present-day eukaryotic cells, these two organelles play pivotal roles in photosynthesis and ATP production. In addition to these major activities, numerous reactions, and cellular processes that are crucial for normal cellular functions occur in chloroplasts and mitochondria. To function properly, these organelles constantly communicate with the surrounding cellular compartments. This communication includes the import of proteins, the exchange of metabolites and ions, and interactions with other organelles, all of which heavily depend on membrane proteins localized to the outer envelope membranes. Therefore, correct and efficient targeting of these membrane proteins, which are encoded by the nuclear genome and translated in the cytosol, is critically important for organellar function. In this review, we summarize the current knowledge of the mechanisms of protein targeting to the outer membranes of mitochondria and chloroplasts in two different directions, as well as targeting signals and cytosolic factors.

## INTRODUCTION

Mitochondria and plastids are two endosymbiotic organelles that have contributed greatly to the evolution of present-day eukaryotic cells. Mitochondria exist in all eukaryotic cell types and are involved in apoptosis, respiratory ATP production, and iron-sulfur cluster assembly (Schleiff and Becker, 2011). By contrast, plastids exist in the plant and in algae and differentiate into multiple subtypes in plants depending on the cell type. Of these plastid-derived organelles, chloroplasts participate in numerous essential metabolic and cellular processes, including photosynthesis, amino acid and lipid metabolism, cell signaling, and host defense (Kessler and Schnell, 2009). According to the endosymbiont hypothesis, mitochondria, and plastids evolved from endosymbiotic bacteria, i.e., α-proteobacterium and cyanobacterium, respectively, in eukaryotic ancestor cells in a sequential manner, with mitochondria evolving first (John and Whatley, 1975; Cavalier-Smith, 2000; Dolezal et al., 2006). During their organellogenesis, one key event was the massive transfer of genetic information from the endosymbionts to the host cell nucleus. Currently, more than 95% of mitochondrial and plastid proteins in present-day eukaryotic cells are encoded by the nuclear genome, synthesized on cytosolic ribosomes, and imported into these organelles (Leister, 2003; Sickmann et al., 2003; Schleiff and Becker, 2011).

Like their ancestors, chloroplasts, and mitochondria contain two envelope membranes that function as chemical and physical barriers to separate organelle-localized metabolic reactions and processes from the cytosol. At the same time, to ensure their organellar functions as part of the cellular system, mitochondria and chloroplasts have evolved a way to communicate with their surroundings at the two envelope membranes, often employing direct physical interactions with other cellular compartments. These communication processes include import of nuclear-encoded proteins and exchanges of metabolites and ions (Inoue, 2011). The nuclear genome encodes all chloroplast and mitochondrial outer membrane and intermembrane space (IMS) proteins as well as most inner membrane and interior proteins (Sato et al., 1999; Neupert and Herrmann, 2007; Schmidt et al., 2010). Of these organellar proteins, outer envelope proteins play crucial roles in many cellular processes such as protein import into organelles, organelle movement and division, and lipid synthesis. These processes are essential not only for the function of their cognate organelles but also for plant development and growth under normal conditions, as well as survival under adverse environmental conditions (Inoue, 2011). Moreover, the mitochondrial outer membrane harbors proteins that control central cellular events such as apoptosis and innate immunity (Walther and Rapaport, 2009).

For all of these processes to occur successfully and efficiently, the specific targeting of organellar proteins to their target must first occur following translation on the cytosolic ribosomes. The outer membrane proteins are a group of heterogeneous proteins that can be divided into multiple types, including α-helical transmembrane domain (TMD)-containing proteins and β-barrel proteins consisting of multiple transmembrane β-strands, based on their structure. Moreover, TMD-containing proteins are further divided into four types, including those with an N-terminal TMD, middle TMD, a C-terminal TMD, and multi-TMDs, according to their topology. These different types of membrane proteins are targeted to their destinations by different mechanisms (Hofmann and Theg, 2005; Walther and Rapaport, 2009; Kim and Hwang, 2013). To understand the targeting of these proteins, it is essential to identify their targeting signals as well as the molecular machinery involved in this targeting. Recently, significant progress has been made in the identification of the targeting signals of membrane proteins (Walther et al., 2009b; Dhanoa et al., 2010; Lee et al., 2011; Weis et al., 2013). By contrast, limited information is available about the machinery used for targeting. In this review, we mainly focus on recent advances in understanding the cytosolic events of protein targeting to the outer envelope membranes in two endosymbiotic organelles in plants, i.e., chloroplasts and mitochondria.

## TARGETING SIGNALS OF CHLOROPLAST AND MITOCHONDRIAL OUTER MEMBRANE PROTEINS

### TARGETING SIGNALS OF CHLOROPLAST AND MITOCHONDRIAL SIGNAL-ANCHORED PROTEINS

Signal-anchored (SA) proteins are a class of membrane proteins that contain a single TMD at their N-terminal regions. SA proteins are involved in important biological processes, functioning as receptors of chloroplast and mitochondrial precursor proteins and biosynthetic enzymes of lipid membranes (Dukanovic and Rapaport, 2011; Inoue, 2011). Mitochondrial and chloroplast SA proteins lack a cleavable targeting sequence, such as a presequence or transit peptide, which mediates specific targeting to the mitochondria or chloroplasts, respectively. Instead, the TMD functions as the targeting signal required for targeting to the correct location, which also occurs with endoplasmic reticulum (ER)-targeted SA proteins (Rapoport, 2007; Schleiff and Becker, 2011). The majority of studies on the targeting mechanisms of mitochondrial SA proteins have been carried out with mammalian and yeast proteins. These studies did not reveal conserved sequence motifs of TMDs that are involved in the determination of targeting specificity. Instead, moderate hydrophobicity of the TMD is one feature that is critical for the mitochondria targeting of SA proteins (Kanaji et al., 2000). When the TMD hydrophobicity of rat Tom20 (rTom20) was increased by introducing more hydrophobic leucine residues, the mutant form of rTom20 was mistargeted to the ER instead of the mitochondrial. Similarly, replacement of the TMD of yeast Tom20 (yTom20) with the more hydrophobic TMD of an ER SA protein inhibited mitochondrial targeting (Waizenegger et al., 2003). The importance of moderate hydrophobic TMD for mitochondrial targeting was confirmed in plant mitochondrial SA proteins in *Arabidopsis* protoplasts (Lee et al., 2011). Intriguingly, the TMDs of chloroplast SA proteins also have moderate hydrophobicity. Increasing the hydrophobicity of the TMD in atToc64 altered its localization from the chloroplast to the plasma membrane (PM; Lee et al., 2004), indicating that the moderately hydrophobic TMD is important for the targeting of SA proteins to both endosymbiotic organelles. However, the concept of moderate hydrophobicity is too ambiguous to be used to differentiate mitochondrial/chloroplast SA proteins from ER SA proteins. A recent study on the targeting of a large number of ER, mitochondrial, and chloroplast SA proteins of *Arabidopsis* revealed that the Wimley and White (WW) hydrophobicity scale is most accurate for differentiating targeting specificity based on the hydrophobicity value of the TMD; more than 85% of all ER SA proteins have a hydrophobicity value greater than 0.4 on the WW hydrophobicity scale, and more than 89% of the mitochondrial and chloroplast SA proteins have hydrophobicity values below 0.4 on the WW hydrophobicity scale (Lee et al., 2011). This rule also applies to most mammalian and yeast mitochondrial SA proteins, suggesting that it applies to all eukaryotic cells. Another critical motif for the targeting of mitochondrial and chloroplast SA proteins is the C-terminal positively charged flanking region (CPR) of the TMD. CPRs usually contain three or more basic residues (arginines and/or lysines) within a short C-terminal flanking region of the TMD (Rapaport, 2003; Lee et al., 2011). Both the moderate hydrophobic TMD and CPR are required for targeting to mitochondria and chloroplasts. Similarly, the basic residues are crucial for the proper targeting of mammalian mitochondrial SA proteins. When basic residues in the CPRs of rTom20 and rTom70 are substituted with serine residues, the mutant proteins are targeted to the ER or Golgi but not to the mitochondria (Kanaji et al., 2000; Suzuki et al., 2002). However, unlike mammalian mitochondrial SA proteins, the CPR is not crucial for mitochondrial targeting in yeast; substitution of basic residues with serines is tolerated (Waizenegger et al., 2003). However, substitution of basic residues with acidic residues inhibits mitochondrial targeting in yeast, indicating that the amino acid composition of the C-terminal flanking region is an important determinant for the targeting specificity of SA proteins (Waizenegger et al., 2003). The CPR is also crucial for the targeting of chloroplast SA proteins. Substitution of basic residues with glycines alters the localization of the chloroplast SA proteins OEP7 and atToc64 to the PM in *Arabidopsis* protoplasts. However, to date, the exact definition of the CPR has not been established. Three basic residues in the CPR are a minimal requirement (Lee et al., 2001, 2004). However, the density of basic amino acid residues in a short C-terminal flanking region appears to be crucial for CPR function. In addition, other factors such as the amino acid composition of the CPR and its distance from the TMD are also important features in defining the CPR. Unlike mammalian cells and yeast, plant cells contain two endosymbiotic organelles, chloroplast, and mitochondria. Therefore, another challenging issue in plants is how they specifically target chloroplast or mitochondrial SA proteins, which have similar targeting signals consisting of the moderately hydrophobic TMD and the CPR.

### TARGETING SIGNALS OF CHLOROPLAST AND MITOCHONDRIAL TAIL-ANCHORED PROTEINS

Tail-anchored (TA) proteins are another class of chloroplast and mitochondrial outer membrane proteins that contain a single TMD at their C-terminal region. Like SA proteins, TA proteins do not harbor a cleavable signal sequence for their targeting (Borgese et al., 2007). The targeting of mitochondrial TA proteins has been studied in mammalian and yeast systems, and these studies have suggested that the targeting signal of mitochondrial TA proteins consists of a short TMD with moderate hydrophobicity and basic residues in the C-terminal sequence (CTS) following the TMD (Rapaport, 2003; Dukanovic and Rapaport, 2011). In plants, the targeting signal of mitochondrial TA proteins has been studied using cytochrome b5 isoforms of tung (*Aleurites fordii*; Hwang et al., 2004). Like mammalian and yeast mitochondrial TA proteins, basic residues in the CTS following the TMD are important for mitochondrial targeting. In addition, the amino acid composition of the TMD is also important for mitochondrial targeting (Hwang et al., 2004).

Many chloroplast TA proteins have been identified (**Table 1**), but the targeting of most of these proteins has not been analyzed in detail. Chloroplast TA proteins do not seem to share any conserved sequence for targeting specificity. A noticeable feature of these proteins is that the hydrophobicity value of TMDs of chloroplast TA proteins appears to vary significantly compared to that of mitochondrial TA proteins containing a moderately hydrophobic TMD. It is possible that the hydrophobicity of TMD is not an important factor for determining chloroplast targeting. Instead, plants have a unique targeting mechanism, as demonstrated for the GTPase domain that acts as a targeting signal of Toc33 and Toc34 (Dhanoa et al., 2010), as described below. In addition, the net charge in the CTS of chloroplast TA proteins does not exhibit any trend, although the basic net charge is an important factor for the proper targeting of mitochondrial TA proteins. Still, basic residues are present in both sides of TMDs to produce a positive net charge. More detailed analysis is required to define the relationship between the net charge in the CTS and the targeting specificity of chloroplast TA proteins.

**Table 1:** *Arabidopsis* proteins in the outer membranes of chloroplasts and mitochondria.

Topology class	Name
**Mitochondrial outer envelope membrane proteins**
Signal-anchored	mtOM64, Hexokinase(s), Pectl, Cbrl (At5g17770), *Atlg53000, *At4g28020
Tail-anchored	FislA, FislB, Tom20(s), Tom5, Tom6, Tom7-1, Tom7-2, Tom9-l, Tom9-2
β -barrel	Tom40, SAM50(s), VDAC(s)
**Chloroplast outer envelope membrane proteins**
Signal-anchored	atTOC64, OEP7, *At4g16070, *At4g27610, *At5g11250
Tail-anchored	Toc33, Toc34, OEP9, PDV1, PDV2, OEP61, Toc159, Cytochrome b5 (Atlg26340)
β -barrel	OEP21, OEP24, OEP37, Toc75, Toc75-V/OEP80, atToc75-IV

*Chloroplast and mitochondrial outer membrane proteins are classified as signal anchored (SA), tail anchored (TA), or β-barrel proteins according to their topology. SA and TA proteins were searched in the UniProtKB database (http://www.uniprot.org/) based on the criteria of experimentally confirmed localization and the existence of a predicted single transmembrane domain. Information about β-barrel proteins was obtained from the literature, which describes their localizations, as determined through proteomic analysis. Proteins indicated by asterisks were not searched in the UniProtKB database but their localizations were confirmed by fluorescence microscopy of GFP-tagged fusion proteins.*

Only a few of these chloroplast TA proteins have been studied in detail. These include Toc33 and Toc34, which are involved in the import of transit peptide-containing precursors into chloroplasts. Toc33 and 34 have two domains, the N-terminal GTPase and the C-terminal TMD; the TMD is involved in anchoring to the outer membrane of the chloroplast. Unlike mitochondrial TA proteins, the C-terminal region including the TMD of Toc33 or 34 is necessary but not sufficient for chloroplast targeting *in vivo* (Dhanoa et al., 2010). In addition to the TMD, the GTPase domain is also necessary for chloroplast localization. Toc159 is another chloroplast TA protein with a GTPase domain; the GTPase domain alone binds to the surface of the chloroplast *in vitro* (Smith et al., 2002). However, given that a truncated form of Toc159 lacking the GTPase domain also binds to the surface of the chloroplast, the role of the GTPase domain in the targeting mechanism of TA proteins seems to be restricted to specific cases, such as Toc33 and Toc34. The GTPase domain of Toc33 interacts with that of Toc159 (Bauer et al., 2002; Smith et al., 2002); thus the interaction between the two GTPase domains has been suggested to be involved in the targeting of Toc33 to the outer membrane of the chloroplast.

The chloroplast targeting mechanism of OEP9 is slightly different from that of Toc33 and Toc34. In the case of OEP9, the TMD and CTS are necessary and sufficient for targeting to the chloroplast (Dhanoa et al., 2010). In fact, replacing the CTS of tung mitochondrial cytochrome b5 with the CTS of OEP9 causes this protein to be targeted to the chloroplast. Similarly, the CTS of OEP9 mediates chloroplast localization of a truncated form of Toc33 lacking the GTPase domain. Analysis of various point mutants has suggested that the net charge or charge distribution in the CTS of OEP9 is crucial for meditating chloroplast targeting (Dhanoa et al., 2010). However, the physical–chemical property of the CTS of OEP9 may not be generally applied to the CTS of other proteins involved in chloroplast targeting. For example, when the CTS of *Arabidopsis* chloroplast cytochrome b5 is replaced with that of tung mitochondrial cytochrome b5, the chimeric form of tung mitochondrial cytochrome b5 is still targeted to the mitochondria (Hwang et al., 2004). The CTS of Toc33 even inhibits the chloroplast targeting of OEP9. These results suggest that the CTSs of *Arabidopsis* chloroplast TA proteins are not always sufficient to support chloroplast targeting, and additional sequence information is necessary depending on the specific protein.

### TARGETING SIGNALS OF CHLOROPLAST AND MITOCHONDRIAL β-BARREL PROTEINS

β-barrel membrane proteins, comprising multiple transmembrane β-strands, are also found in chloroplast and mitochondrial outer membranes (Walther et al., 2009b). These proteins are involved in transporting metabolites, ions, or precursor proteins at the outer membranes of chloroplasts and mitochondria. Mitochondrial β-barrel proteins do not contain cleavable targeting sequences involved in delivery to the mitochondria from the cytosol after translation (Rapaport, 2003). The targeting signal is not restricted to a specific region but is dispersed throughout the polypeptide sequence (Court et al., 1996; Rapaport and Neupert, 1999). Based on these features, it has been proposed that the targeting information is contained in the secondary and/or tertiary structures rather than in a specific sequence motif of the primary sequence (Walther et al., 2009b). Interestingly, bacterial β-barrel proteins are targeted to, and properly inserted into, the outer membrane of mitochondria in yeast (Walther et al., 2009a). This result suggests that the mitochondrial targeting information of β-barrel proteins has been derived from that of the ancestral β-barrel proteins.

Chloroplasts also contain β-barrel proteins in the outer envelope membrane. Toc75 is a chloroplast β-barrel protein that functions as the channel for translocation of transit peptide-containing chloroplast precursor proteins across the outer membrane (Tranel and Keegstra, 1996). Unlike mitochondrial β-barrel proteins, Toc75 contains a cleavable targeting sequence, the transit peptide that is essential for targeting to the chloroplast after translation in the cytosol (Tranel and Keegstra, 1996). Another β-barrel protein, Toc75-V/OEP80, an isoform of Toc75, was also predicted to have a transit peptide at its N-terminus. However, the N-terminal region of Toc75-V/OEP80 is dispensable for chloroplast targeting (Patel et al., 2008). Similarly, other β-barrel proteins such as OEP24 and OEP37, but not OEP21, were predicted to have transit peptides; however, whether the predicted transit peptides are involved in chloroplast targeting remains to be experimentally confirmed. Overall it remains largely elusive if the targeting of β-barrel proteins to chloroplasts is similar to that to mitochondria.

**FIGURE 1:**
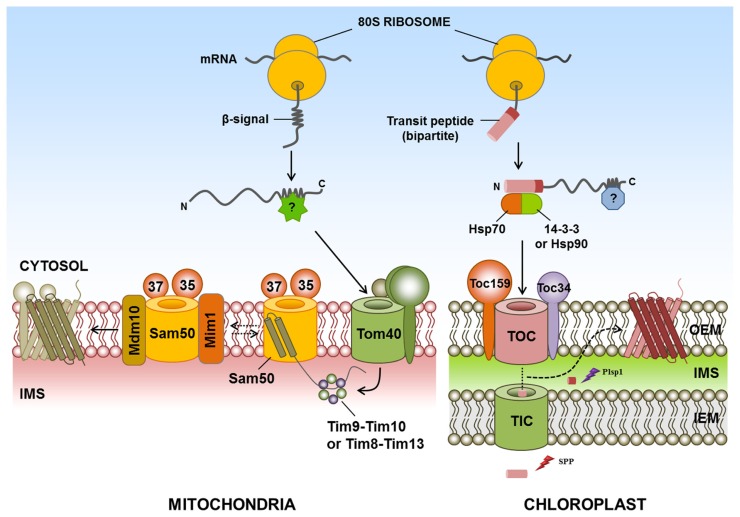
**Targeting of β-barrel proteins to the chloroplast and mitochondrial outer membranes.** Specific cytosolic factors for targeting of β-barrel proteins of the chloroplast and mitochondria have not yet been identified in plants, mammals, or yeast. The chloroplast outer membrane-targeted β-barrel proteins Toc75, OEP24, and OEP37 have (or are predicted to have) typical transit peptides. For chloroplast targeting, Toc75 requires an N-terminal transit peptide comprising two parts. The first part of the targeting signal is cleaved in the stroma by SPP, whereas the second part is removed by Plsp1. However, the exact mechanism of insertion of β-barrel proteins into the chloroplast outer membrane is still unknown. In the mitochondrial outer membrane-targeted β-barrel proteins, cytosolic β-barrel precursors are recognized by the TOM complex and translocated into the IMS by Tom40. IMS chaperone complexes Tim9-Tim10 and Tim8-Tim13 bind to the β-barrel proteins and transfer these proteins to the SAM complex. Subsequently, Sam50, Sam35, and Sam37 complexes promote the release of β-barrel proteins into the lipid phase of the outer membrane. In the case of β-barrel proteins, Mim1 promotes the assembly of Tom40 by transiently binding to the SAM complex. In addition, Mdm10 may promote the assembly of the TOM complex. IMS, intermembrane space; Mdm10, mitochondrial distribution and morphology 10; Mim1, mitochondrial import 1; Plsp1, plastidic type I signal peptidase 1; SAM, sorting and assembly machinery; SPP, stromal processing peptidases; postulated factor; dashed arrow, postulated pathway; ?, postulated factor.

Most functional studies on the biogenesis of β-barrel proteins have been performed on bacteria, mammals and yeast but, unfortunately, specific cytosolic components for the sorting and targeting of β-barrel proteins of chloroplasts or mitochondria have not yet been identified in plants, mammals or yeast. Intriguingly, with the exception of Toc75, all outer membrane proteins identified to date, including β-barrel proteins in the chloroplast and mitochondria, are synthesized at the mature size without a cleavable signal sequence (Rapaport, 2003; Hofmann and Theg, 2005; Chacinska et al., 2009; Li and Chiu, 2010; Schleiff and Becker, 2011). Chloroplast-targeted Toc75 has an N-terminal signal sequence consisting of two parts. The first part is a typical transit peptide, whereas the second part comprises a region rich in hydrophobic residues and a polyglycine stretch (Inoue and Keegstra, 2003). The first part of the targeting signal is cleaved in the stroma by stromal processing peptidase, whereas the second part is removed by a membrane-bound peptidase known as plastidic type I signal peptidase 1 (Inoue et al., 2005; **Figure 1**). However, the exact mechanism of its insertion into the chloroplast outer membrane is still unknown.

In the case of the yeast mitochondria, β-barrel proteins are initially recognized by the TOM complex, consisting of Tom20 and Tom70. These proteins are then translocated into the IMS through the import channel Tom40. In the IMS, the chaperone complexes Tim9-Tim10 and Tim8-Tim13 bind to β-barrel proteins (Hoppins and Nargang, 2004; Wiedemann et al., 2004) and participate in the transport of these proteins to the sorting and assembly machinery (SAM complex) on the mitochondrial outer membrane (Paschen et al., 2003; Wiedemann et al., 2003). The β-barrel proteins are inserted into a hydrophilic environment within the SAM complex. These proteins subsequently bind to Sam35 and Sam37, two partner proteins of Sam50, which promotes the release of the β-barrel proteins into the lipid phase of the outer membrane (Paschen et al., 2003; Wiedemann et al., 2003; Gentle et al., 2004; Chan and Lithgow, 2008; Kutik et al., 2008). Mitochondrial import 1 (Mim1) in yeast, which is involved in the membrane insertion of mitochondrial SA protein, also promotes the assembly of β-barrel proteins by transiently binding to the SAM complex, and this protein may modulate SAM function (Ishikawa et al., 2004; Becker et al., 2008; Popov-Celeketic et al., 2008; Chacinska et al., 2009). Moreover, Mdm10 in yeast may promote the assembly of the TOM complex (Boldogh et al., 2003; Meisinger et al., 2007; **Figure 1**); however, the exact mechanism of the insertion and release of β-barrel proteins is not yet known.

## CYTOSOLIC FACTORS OF CHLOROPLAST AND MITOCHONDRIAL OUTER MEMBRANE PROTEINS

### CYTOSOLIC TARGETING FACTORS FOR CHLOROPLAST AND MITOCHONDRIAL SIGNAL-ANCHORED PROTEINS

**FIGURE 2:**
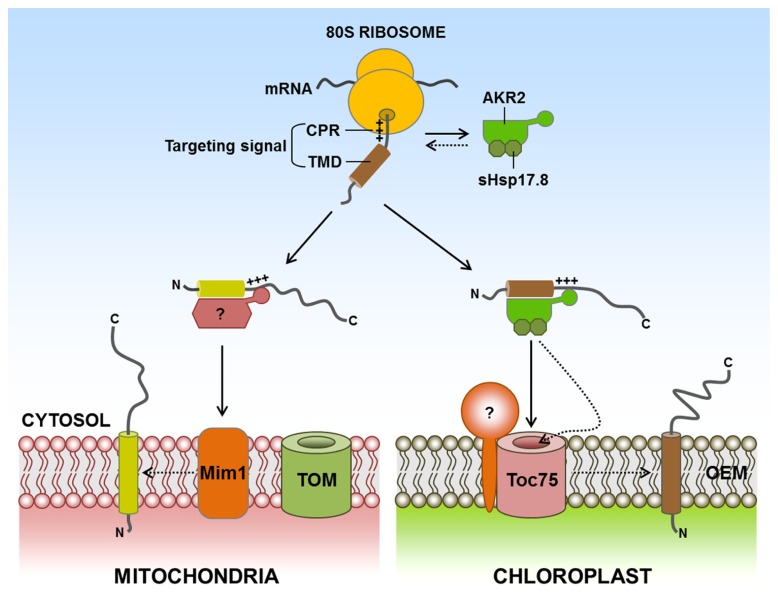
**Targeting of signal-anchored (SA) proteins to the outer membranes of chloroplasts and mitochondria.** SA proteins contain a TMD that functions as both an anchor to outer envelope membranes and a targeting signal. In addition, the CPR functions as an ER-evading signal, which is necessary for endosymbiotic organellar targeting. For targeting of SA proteins to the chloroplast outer membrane, AKR2 interacts with a targeting signal consisting of TMD and CPR and delivers the proteins to the chloroplast outer membrane. The activity of AKR2 is assisted by the Class I small heat shock family protein sHsp17.8. Interestingly, sHsp17.8 (as a dimer) binds to both AKR2 and chloroplasts. However, no cytosolic factors have been identified for targeting of mitochondrial outer membrane SA proteins. Mim1 is involved in the insertion of certain mitochondrial outer membrane SA proteins into the mitochondrial outer membrane. AKR2, ankyrin-repeat protein 2; CH, chloroplast; CPR, C-terminal positively charged flanking region; ER, endoplasmic reticulum; Mim1, mitochondrial import 1; TMD, transmembrane domain; +++, positively charged amino acid residues; ?, postulated factor; dashed arrow, postulated pathway.

Chloroplast and mitochondrial SA proteins are targeted post-translationally. Currently, an important question is whether any cytosolic factor(s) play(s) a role in the targeting of SA proteins from the cytosol to these organelles. In the case of protein targeting to the ER in eukaryotes, signal recognition particles (SRPs) mediate this targeting in a co-translational manner (Keenan et al., 2001). Recently, ankyrin-repeat-containing protein 2, consisting of two isoforms, AKR2A and AKR2B, has been identified as a cytosolic factor for targeting of SA proteins to the chloroplast outer membrane (Bae et al., 2008; Bédard and Jarvis, 2008; Kim et al., 2011). AKR2A interacts with the targeting signals of chloroplast outer membrane proteins (consisting of the TMD and the CPR) *in vitro* and *in vivo*, but not the targeting signals of proteins destined for endomembrane organelles (Lee et al., 2001, 2004). Additionally, AKR2A displays chaperone activity and prevents non-specific aggregation of its client proteins by binding to the hydrophobic TMD. Chaperone activity should be an integral part of cytosolic targeting factors for the post-translational targeting of membrane proteins because these factors can use this activity to keep their clients in an insertion-competent form in the cytosol by preventing non-specific aggregate formation, proteolytic degradation, or unproductive interactions with other proteins before organellar membrane proteins are delivered to the target membranes (Flores-Pérez and Jarvis, 2013; Kim and Hwang, 2013). In addition, AKR2 binds to chloroplasts through its C-terminal ankyrin-repeat domain (ARD) and facilitates insertion of its client proteins into the chloroplast outer membrane, where Toc75 assists with their insertion (Tu et al., 2004). Recently, it has been shown that AKR2 is associated with sHsp17.8, a member of the cytosolic Class I small heat shock protein (sHsp) family (Kim et al., 2011; **Figure 2**). Interestingly, sHsp17.8 (as a dimer) binds to both AKR2 and chloroplasts. Through these interactions, sHsp17.8 facilitates AKR2-mediated targeting of SA proteins to the chloroplast outer membrane, suggesting that sHsp17.8 functions as a cofactor of AKR2 during protein targeting to the chloroplast outer membrane.

It remains elusive when and how AKR2 specifically recognizes its SA clients in the cytosol during protein targeting to the chloroplast outer membrane. When nascent organellar proteins emerge from the exit tunnel of ribosomes during translation, they may interact with specific targeting factors and/or chaperones that assist in targeting to the proper location of the cell (Ullers et al., 2003; Schlünzen et al., 2005; Spreter et al., 2005). In the case of the ER in mammalian cells and yeast, the SRP recognizes the hydrophobic signal sequence of ER luminal and SA proteins during translation (Keenan et al., 2001). Moreover, Bat3/TRC35/Ubl4A complexes, which are pre-targeting factors for TA proteins of the ER in mammalian cells, also associate with ribosomes (Fleischer et al., 2006; Jonikas et al., 2009; Mariappan et al., 2010), suggesting that ribosomes serve as a platform for the docking of cytosolic factors involved in organellar protein targeting. These studies raise the possibility that AKR2 recognizes the chloroplast outer membrane-targeted SA proteins at the ribosomes during translation. Additionally, another important question is how AKR2 recognizes chloroplasts as the target organelle. The C-terminal ARD of AKR2 is involved in chloroplast binding, and its binding is assisted by sHsp17.8 (Bae et al., 2008; Kim et al., 2011). However, in the absence of sHsp17.8, AKR2A still binds to the chloroplast *in vitro*, raising the possibility that AKR2A alone interacts with the chloroplast. This result strongly suggests that certain factors exist on the chloroplast outer membrane for AKR2 recruitment.

For precursor protein import into endosymbiotic organelles, many soluble factors in plants, mammalian cells and yeast have been identified that include Hsp70, Hsp90, 14-3-3 proteins, mitochondrial stimulating factor (MSF), arylhydrocarbon receptor-interacting protein (AIP), nascent polypeptide-associated complex (NAC), and ribosome-associated complex (RAC; Hachiya et al., 1994; Fünfschilling and Rospert, 1999; May and Soll, 2000; Gautschi et al., 2001; Yano et al., 2003; Young et al., 2003; Qbadou et al., 2006; Schemenewitz et al., 2009). These cytosolic factors with a chaperone activity may play an important role in keeping preproteins in import-competent status by preventing their aggregation or degradation, or minimizing unproductive interactions with other proteins in the cytosol (Flores-Pérez and Jarvis, 2013; Kim and Hwang, 2013). Hsp70 can act alone or in cooperation with other soluble factors such as Hsp90, 14-3-3 proteins or AIP (in mammalian), and these factors may also play a role in facilitating delivery of preproteins to receptors localized on the surface of chloroplast or mitochondrial outer membranes (Hachiya et al., 1994; May and Soll, 2000; Yano et al., 2003; Young et al., 2003; Qbadou et al., 2006; Schemenewitz et al., 2009). In yeast or mammalian cells, NAC, RAC, AIP, and MSF can stimulate the import of preproteins into mitochondria (Hachiya et al., 1994; Fünfschilling and Rospert, 1999; Gautschi et al., 2001; Yano et al., 2003). Currently, no cytosolic factors have been identified for targeting mitochondrial outer membrane SA proteins. They also contain a hydrophobic TMD that serves as an anchor to the outer membrane and also functions as a targeting signal. Therefore, a mechanism should exist that solves the problem of non-specific aggregate formation of the hydrophobic TMD of mitochondrial outer membrane SA proteins in the aqueous cytosol, raising the possibility that a yet unidentified factor(s) may be involved in the delivery of SA proteins to the mitochondria. Additional information is available about protein factors that are involved in the steps that occur at the mitochondrial outer membrane. Mim1 in yeast is involved in the insertion of certain SA proteins into the mitochondrial outer membrane (Becker et al., 2008; Hulett et al., 2008; Popov-Celeketic et al., 2008; Chacinska et al., 2009; **Figure 2**). Mim1 catalyzes the docking step for an α-helical transmembrane segment of Tom20 and Tom70 onto a membrane protein complex formed around a β-barrel protein Tom40 (Becker et al., 2008; Hulett et al., 2008). Mim1 forms homodimers in the mitochondrial outer membrane via its transmembrane segment, which contains two consecutive GXXXG/A motifs. The two GXXXG/A motifs are crucial for formation of dimers and also for integration of Tom20 into the mitochondrial outer membrane (Popov-Celeketic et al., 2008). In addition, the core of the TOM complex, Tom40, is involved in the insertion of certain SA proteins into the mitochondria in yeast (Ahting et al., 2005). However, most SA proteins do not seem to require the TOM complex for this insertion (Schneider et al., 1991; Schlossmann and Neupert, 1995; Ahting et al., 2005).

### CYTOSOLIC TARGETING FACTORS FOR CHLOROPLAST AND MITOCHONDRIAL TAIL-ANCHORED PROTEINS

The location of the hydrophobic TMD poses an additional complication during the targeting of TA proteins because the TMD must be recognized post-translationally (Chartron et al., 2012). For ER-localized TA proteins, the guided entry of the TA protein (GET) pathway in yeast is used to deliver these proteins to the ER membrane. This pathway starts with the transfer of TA proteins from ribosomes to the “pre-targeting” factor (Get4/Get5/Sgt2), the sorting complex, which then loads them onto the targeting factor Get3 (Mariappan et al., 2010; Wang et al., 2010; Chartron et al., 2012). The central protein Get3 utilizes nucleotide-linked conformational changes in the loading and targeting of client proteins; a “closed” dimer of Get3 binds to the TA client via its large hydrophobic groove. The GET3-client complex is recruited to the ER membrane by binding to the Get1/Get2 receptor complex.

**FIGURE 3:**
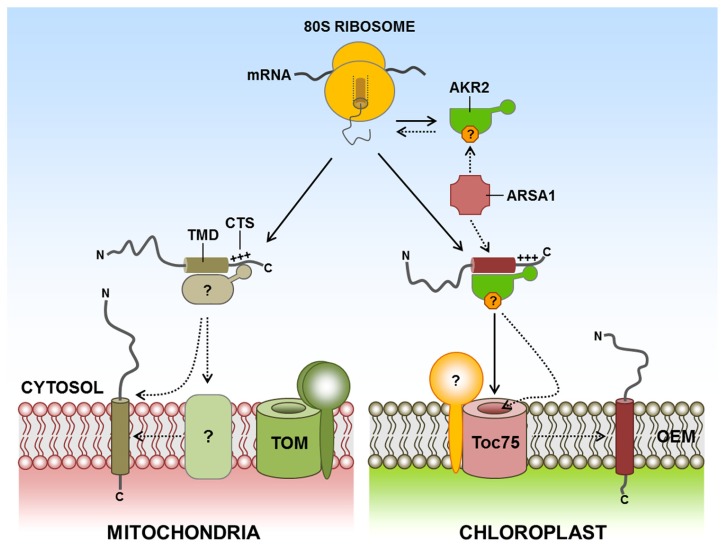
**Targeting of TA proteins to the chloroplast and mitochondrial outer membranes.** TA proteins are characterized by a C-terminal single TMD and the CTS. However, little information is available about the molecular machinery and mechanisms of the targeting of TA proteins to the outer membranes of chloroplasts and mitochondria. AKR2 also binds to chloroplast outer membrane TA proteins such as OEP9, Toc33, and Toc34. In addition, the cytosolic factor ARSA1 mediates the biogenesis and targeting of Toc34 from the cytosol to the chloroplast outer membrane. In the case of mitochondria, no cytosolic component for outer membrane-targeted TA proteins has yet been identified. However, the unique lipid composition of the outer membrane is important for the efficient insertion of TA proteins to the mitochondria. AKR2, ankyrin-repeat protein 2; ARSA1, arsenite transport 1; CTS, C-terminal sequence; TA, tail-anchored; TMD, transmembrane domain; +++, positively charged amino acid residues; ?, postulated factor; dashed arrow, postulated pathway.

By contrast to the ER targeting of TA proteins, little information is available about the molecular machinery and mechanisms of TA protein targeting to the chloroplast and mitochondrial outer membranes. Chloroplast outer membrane TA proteins such as OEP9 and Toc33/Toc34 also interact with the cytosolic targeting factor AKR2, which is involved in targeting SA proteins to the chloroplast outer membrane (Bae et al., 2008; Dhanoa et al., 2010). However, the targeting signal of Toc33/Toc34 is different from that of OEP9. In the case of OEP9, the targeting signal consists of a 32 amino acid-long hydrophilic CTS and the TMD (Dhanoa et al., 2010). Toc33 and Toc34 also have single TMDs at their C-terminal ends, followed by CTS. However, their targeting to the chloroplast outer membrane depends on almost the entire protein sequence rather than TMD and CTS, raising the possibility that the targeting of TA proteins to the chloroplast outer membrane may not solely depend on AKR2. Recently, an arsenite transporter, ARSA1, has been identified as a cytosolic factor that mediates biogenesis and targeting of Toc34 from the cytosol to the chloroplast outer membrane in *Chlamydomonas reinhardtii* (Formighieri et al., 2013). Intriguingly, TRC40 and GET3 are also homologs of ARSA1 in mammalian cells and yeast, respectively. By contrast to ARSA1, these proteins are involved in the targeting of TA proteins to the ER membrane. Interestingly, only one ARSA1 homolog gene has been found in humans and yeast. However, two and three ARSA homolog genes are present in *C. s reinhardtii* and *Arabidopsis*, respectively (Chartron et al., 2012; Formighieri et al., 2013; **Figure 3**). Since ARSA1 is involved in the targeting of chloroplast proteins, other ARSA homologs may be involved in targeting TA proteins to the ER in plants, which is similar to that in animals and yeast. However, it is not clear whether AKR2 is involved in the targeting of TA proteins to the chloroplast, or whether AKR2 communicates with ARSA homologs for targeting TA proteins to the chloroplast outer membrane in plants. On the other hand, certain chloroplast TA proteins translated in wheat germ extracts were efficiently targeted to chloroplast outer membranes with high fidelity (Kriechbaumer and Abell, 2012). Based on these results, they suggested that cytosolic factors may play a minor role, if there is any, in TA protein targeting to chloroplasts, and that targeting of proteins to chloroplast envelope membrane is primarily dependent on events at the outer membrane. However, in the *in vitro* assay systems, it is difficult to discriminate between nonspecific association of the hydrophobic transmembrane segment with the membrane and physiological membrane integration (Borgese et al., 2003). In the cellular environment, cytosolic targeting factors may be required to protect the targeting signal including hydrophobic TMD and to keep nascent TA proteins in insertion-competent status by preventing their aggregation, or minimizing unproductive interactions with other proteins in the cytoplasm (Ellis and Minton, 2006; Flores-Pérez and Jarvis, 2013; Kim and Hwang, 2013).

In the case of mitochondrial outer membrane TA proteins, a bioinformatics approach has predicted that 142 out of 454 TA proteins in *Arabidopsis* may localize to the mitochondria (Kriechbaumer et al., 2009). Limited information is available about the molecular machinery that directs C-terminal α-helical TMD-containing proteins into the mitochondrial outer membrane from the cytosol. To date, no cytosolic components have been identified. Although the TOM complex or Mim1 appear to be required for the insertion of certain mitochondrial outer membrane TA proteins (Motz et al., 2002; Thornton et al., 2010), these mitochondrial TA proteins may use an unidentified insertase, or they may be spontaneously inserted into the outer membrane of the mitochondria. However, in Bak and Bcl-XL in mammalian cells, and Fis1 in yeast, the C-terminal transmembrane segment is sufficient for mitochondrial outer membrane targeting and none of the import components at the outer membrane is involved in the insertion (Setoguchi et al., 2006; Kemper et al., 2008). Interestingly, the unique lipid composition of the mitochondrial outer membrane appears to contribute to the selectivity in the targeting of membrane proteins (Kemper et al., 2008). In fact, the unique lipid composition of the membrane has been shown to be important for efficient insertion of TA proteins into the mitochondria (Setoguchi et al., 2006; **Figure 3**). However, targeting of full length Bak and Bcl-XL in mammalian cells requires cytosolic factor(s) with a chaperone activity, suggesting that the targeting is critically dependent on the folding status of the N-terminal cytosolic domains (Setoguchi et al., 2006).

## CONCLUDING REMARKS

In this review, we summarized the mechanisms of protein targeting to the outer envelope membranes of two endosymbiotic organelles, chloroplasts, and mitochondria. Proteins localized to the outer membranes of these two organelles are involved in various functions that are essential for the physiology of these organelles. Numerous studies have contributed to our understanding of various aspects of the physiological roles of these organelles. One of these aspects involves how proteins are specifically targeted to these endosymbiotic organelles, and we currently have a fairly good understanding of how proteins are targeted to these organelles at the molecular level. Owing to studies of mitochondrial protein biogenesis in animal cells and yeast, more is known about mitochondria targeting than chloroplast targeting, yet despite this progress, there are still many aspects that we do not fully understand at the molecular level. These include how sorting of the organellar proteins occurs in the cytosol, how these proteins navigate to their specific organelles, and how the proteins are inserted into these organelles. The answers to these questions will help elucidate how organellar protein biogenesis occurs at the molecular level and how these organelles function in eukaryotic cells. Moreover, answering these questions may also provide clues to one of the most challenging and intriguing questions about these organelles, that is, how specific targeting mechanisms have been established during the organellogenesis of chloroplasts and mitochondria. It is likely that the cellular environment of the host cells at the time of their organellogenesis has been incorporated into the targeting mechanisms; thus the targeting signals and molecular machinery involved in the targeting of chloroplast and mitochondrial outer membrane proteins in present-day eukaryotic cells may contain “molecular fossils” that provide insights into how organellogenesis of these two organelles has occurred during evolution.

## Conflict of Interest Statement

The authors declare that the research was conducted in the absence of any commercial or financial relationships that could be construed as a potential conflict of interest.
